# Bone marrow cell therapy in cardiovascular disease drives us slowly to a better identification of the active cell component

**DOI:** 10.1186/scrt405

**Published:** 2014-01-27

**Authors:** David M Smadja

**Affiliations:** 1 Université Paris Descartes, Sorbonne Paris Cité, Faculté de Pharmacie, 75006, Paris, France; 2 Inserm UMR-S1140, 4 avenue de l’observatoire, 75006, Paris, France; 3 AP-HP, Hôpital Européen Georges Pompidou, Hematology Department, 20 rue Leblanc, 75015, Paris, France

## Abstract

Endothelial progenitor cell therapy and stem cell therapy have been proposed in regeneration of acute myocardial infarction (AMI). In the previous issue of *Stem Cell Research & Therapy*, Lamirault and colleagues described a strong analysis of progenitors in blood and bone marrow of patients collected after AMI, and correlated these levels to bone marrow mononuclear cell (BM-MNC) therapy efficacy and smoking status. The main results are that BM-MNCs can override smoking alteration in endothelial lineage and confirm that endothelial progenitor cells are probably not by themselves the active component of BM-MNC in AMI. This paper allows one to better appreciate the cellular complexity of cell therapy approach in AMI.

## 

Endothelial progenitor cell (EPC) therapy and stem cell therapy have been proposed in regeneration of myocardial infarction and peripheral arterial disease. However, there remains an ongoing need to understand what could be the cellular active component appropriate for each clinical situation to make this cell therapy approach impossible to circumvent. In the previous issue of *Stem Cell Research & Therapy*, Lamirault and colleagues described a strong analysis of hematopoietic progenitors and EPC in blood and bone marrow (BM) of patients collected after acute myocardial infarction (AMI), and correlated these levels with bone marrow mononuclear cell (BM-MNC) therapy efficacy and smoking status [1].

The first insight from this study is the specific impairment of the endothelial lineage in active smokers. Indeed, the authors described BM activation during the days following AMI whereas EPC numbers were found lower in active smokers, suggesting a specific lack of activation of the endothelial lineage [1]. This is not the first description of a specific EPC impairment and/or lack of mobilization. Indeed, our group in 2008 described a defect in EPC mobilization in the context of extracorporeal circulation in patients having numerous cardiovascular risk factors [2], while the hematopoietic lineage was strongly enhanced whatever the risk factor status.

These results are not in favor of a mixed lineage between hematopoietic and endothelial lineages and the hemangioblast existence in adults. Indeed, EPCs have been described as clonally different from hematopoietic progenitors in patients with myeloproliferative disorders [3]. This different ontogeny and the presence of clonogenic endothelial cells extracted from umbilical cord blood or lung led Yoder to suggest that EPCs could have a vascular origin [3]. This hypothesis led us to think that EPCs could be efficiently mobilized after vascular stimulation induced by local ischemia. However, because local ischemia does not mobilize any subtypes of EPCs [4], the origin and regulation of EPCs remain controversial and not fully understood. Finally, we have to carefully conclude that since EPCs isolated from smokers, despite the same level in blood as nonsmokers, have increased DNA damage and senescence [5]. This could explain the discrepancy between EPC numbers and an absence of correlation with efficacy.

The second highlight of this study is that cardiac BM cell therapy overrides EPC impairment observed in AMI patients. The cell subset responsible for the beneficial effects of BM cell therapy are not yet identified, but a multitude of studies have been published and have demonstrated that intracoronary BM‒MNC delivery led to a left ventricular ejection fraction (LVEF) improvement. However, most of these trials did not have real biological exploration in parallel to BM‒MNC therapy and several remaining questions exist about the active component and patient’s eligible for BM cell therapy.

Although Lamirault and colleagues’ results shows an absence of correlation between the EPC level and improvement after BM-MNC injection that is descriptive, the findings from this study provide strong evidence that, despite EPC involvement in physiological regeneration due to correlation with the LVEF in placebo group patients, EPCs are probably not the cell type responsible for clinical improvement in the BM-MNC therapy approach. This result is in accordance with several previous hypotheses. First, strong data from preclinical models suggest that BM-MNCs do not differentiate into endothelial cells, and that their paracrine effect is most probably responsible for the angiogenic process [6]. Second, the correlation previously described with BM-MNC efficacy has been achieved with angiogenic cells not able to build vessels by themselves and different from real vasculogenic EPCs [7]. Thus, the more we know about EPCs, the more evidence seems to discredit them as the sole active component.

A recent meta-analysis found that younger patients and patients with a more severely depressed LVEF at baseline benefit from this adjunctive therapy and also 5 to 7 days after AMI produced a superior improvement in global cardiac function (LVEF) when compared with an earlier or delayed delivery time [8]. However, the time lag of a few days between myocardial infarction and treatment excludes the preparation of cell therapy products from cells purified following expansion *in vitro* as mesenchymal stem cells or EPCs. Mesenchymal stem cells induce blood flow recovery *in vivo* to the same degree as in healthy controls, in contrast to the reduced ability that has been described for BM-MNCs and EPCs from patients with cardiovascular disorders [9] – thus mesenchymal stem cells should be a good candidate for cell therapy. So, despite the observation that BM‒MNCs secrete lower amounts of angiogenic and anti-apoptotic growth factors than the mesenchymal stem cell subtype [10], BM-MNCs are probably the best cell therapy product for AMI.

In conclusion, Lamirault and colleagues fill a gap in our knowledge by identifying in a large biological study associated with a cell therapy trial that BM-MNCs can override smoking alteration in endothelial lineage. This result allows us to confirm that EPCs are probably not by themselves the active component of BM-MNCs in AMI, and contributes to better appreciation of the cellular complexity of the cell therapy approach in AMI.

## Abbreviations

AMI: Acute myocardial infarction; BM: Bone marrow; BM-MNC: Bone marrow mononuclear cell; EPC: Endothelial progenitor cell; LVEF: Left ventricular ejection fraction.

## Competing interests

The author declares that he has no competing interests.

## References

[B1] Lamirault G Susen S Forest V Hemont C Parini A Le Corvoisier P Piot C Richard MJ Delasalle B Rouard H Sportouch C Persoons V Van Belle E Roncalli J Lemarchand P Difference in mobilization of progenitor cells after myocardial infarction in smoking vs. non-smoking patients: insights from the BONAMI trial Stem Cell Res Ther 2014 4 152 24423369 10.1186/scrt382 PMC4054959

[B2] Smadja DM Godier A Susen S Packard RR Fabiani JN Aiach M Gaussem P Endothelial progenitor cells are selectively mobilised immediately after coronary artery bypass grafting or valve surgery Thromb Haemost 2009 101 983 985 19404556

[B3] Yoder MC Is endothelium the origin of endothelial progenitor cells? Arterioscler Thromb Vasc Biol 2010 30 1094 1103 10.1161/ATVBAHA.109.191635 20453169

[B4] Mauge L Sabatier F Boutouyrie P D'Audigier C Peyrard S Bozec E Blanchard A Azizi M Dizier B Dignat-George F Gaussem P Smadja DM Forearm ischemia decreases endothelial colony-forming cell angiogenic potential Cytotherapy 2013 [Epub ahead of print] 10.1016/j.jcyt.2013.09.007 24280239

[B5] Paschalaki KE Starke RD Hu Y Mercado N Margariti A Gorgoulis VG Randi AM Barnes PJ Dysfunction of endothelial progenitor cells from smokers and COPD patients due to increased DNA damage and senescence Stem Cells 2013 31 2813 2826 10.1002/stem.1488 23897750 PMC4377082

[B6] Dudley AC Udagawa T Melero-Martin JM Shih SC Curatolo A Moses MA Klagsbrun M Bone marrow is a reservoir for proangiogenic myelomonocytic cells but not endothelial cells in spontaneous tumors Blood 2010 116 3367 3371 10.1182/blood-2010-02-271122 20453162 PMC2995361

[B7] Walter DH Krankenberg H Balzer JO Kalka C Baumgartner I Schluter M Tonn T Seeger F Dimmeler S Lindhoff-Last E Zeiher AM Intraarterial administration of bone marrow mononuclear cells in patients with critical limb ischemia: a randomized-start, placebo-controlled pilot trial (PROVASA) Circ Cardiovasc Interv 2011 4 26 37 10.1161/CIRCINTERVENTIONS.110.958348 21205939

[B8] Delewi R Hirsch A Tijssen JG Schachinger V Wojakowski W Roncalli J Aakhus S Erbs S Assmus B Tendera M Goekmen Turan R Corti R Henry T Lemarchand P Lunde K Cao F Huikuri HV Sürder D Simari RD Janssens S Wollert KC Plewka M Grajek S Traverse JH Zijlstra F Piek JJ Impact of intracoronary bone marrow cell therapy on left ventricular function in the setting of ST-segment elevation myocardial infarction: a collaborative meta-analysis Eur Heart J 2013 [Epub ahead of print] 10.1093/eurheartj/eht372 PMC4271100 24026778

[B9] Smadja DM d'Audigier C Guerin CL Mauge L Dizier B Silvestre JS Dal Cortivo L Gaussem P Emmerich J Angiogenic potential of BM MSCs derived from patients with critical leg ischemia Bone Marrow Transplant 2012 47 997 1000 10.1038/bmt.2011.196 21986637

[B10] Li TS Cheng K Malliaras K Smith RR Zhang Y Sun B Matsushita N Blusztajn A Terrovitis J Kusuoka H Marbán L Marbán E Direct comparison of different stem cell types and subpopulations reveals superior paracrine potency and myocardial repair efficacy with cardiosphere-derived cells J Am Coll Cardiol 2012 59 942 953 10.1016/j.jacc.2011.11.029 22381431 PMC3292778

